# Activations of Both Extrinsic and Intrinsic Pathways in HCT 116 Human Colorectal Cancer Cells Contribute to Apoptosis through p53-Mediated ATM/Fas Signaling by *Emilia sonchifolia* Extract, a Folklore Medicinal Plant

**DOI:** 10.1155/2012/178178

**Published:** 2012-02-28

**Authors:** Yu-Hsuan Lan, Jo-Hua Chiang, Wen-Wen Huang, Chi-Cheng Lu, Jing-Gung Chung, Tian-Shung Wu, Jia-Hua Jhan, Kuei-Li Lin, Shu-Jen Pai, Yu-Jen Chiu, Minoru Tsuzuki, Jai-Sing Yang

**Affiliations:** ^1^School of Pharmacy, China Medical University, Taichung 404, Taiwan; ^2^Department of Life Sciences, National Chung Hsing University, Taichung 402, Taiwan; ^3^Department of Biological Science and Technology, China Medical University, Taichung 404, Taiwan; ^4^Department of Pharmacy, Da Chien General Hospital, Miaoli 360, Taiwan; ^5^Department of Radiation Oncology, Chi Mei Medical Center, Tainan 710, Taiwan; ^6^Department of Medical Education, Far Eastern Memorial Hospital, New Taipei 220, Taiwan; ^7^Department of Biochemistry, Nihon Pharmaceutical University, Saitama 362-0806, Japan; ^8^Tsuzuki Institute for Traditional Medicine, China Medical University, Taichung 404, Taiwan; ^9^Department of Pharmacology, China Medical University, Taichung 40402, Taiwan

## Abstract

*Emilia sonchifolia* (L.) DC (Compositae), an herbaceous plant found in Taiwan and India, is used as folk medicine. The clinical applications include inflammation, rheumatism, cough, cuts fever, dysentery, analgesic, and antibacteria. The activities of *Emilia sonchifolia* extract (ESE) on colorectal cancer cell death have not been fully investigated. The purpose of this study explored the induction of apoptosis and its molecular mechanisms in ESE-treated HCT 116 human colorectal cancer cells *in vitro*. The methanolic ESE was characterized, and *γ*-humulene was formed as the major constituent (63.86%). ESE induced cell growth inhibition in a concentration- and time-dependent response by MTT assay. Apoptotic cells (DNA fragmentation, an apoptotic catachrestic) were found after ESE treatment by TUNEL assay and DNA gel electrophoresis. Alternatively, ESE stimulated the activities of caspase-3, -8, and -9 and their specific caspase inhibitors protected against ESE-induced cytotoxicity. ESE promoted the mitochondria-dependent and death-receptor-associated protein levels. Also, ESE increased ROS production and upregulated the levels of ATM, p53, and Fas in HCT 116 cells. Strikingly, p53 siRNA reversed ESE-reduced viability involved in p53-mediated ATM/Fas signaling in HCT 116 cells. In summary, our result is the first report suggesting that ESE may be potentially efficacious in the treatment of colorectal cancer.

## 1. Introduction

Colorectal cancer is a major reason of death worldwide [1], and it is the third most frequent cause of cancer death in Taiwan. About 20.2 per 100,000 people died of colorectal cancer according to the reports of the Department of Health, R.O.C. (Taiwan) in 2010 (http://www.doh.gov.tw/EN2006/index_EN.aspx/). Recently, various studies have shown that traditional Chinese medicine (TCM) and folklore medicine possessed potential anticolorectal cancer activity and they are associated with a reduced risk of cancer [2–4]. It has been reported that the TCM and folklore medicine can induce reactive oxygen species (ROS) production and DNA damage, which leads to increased phosphorylation of ataxia-telangiectasia-mutated kinase (ATM) and p53 and then triggers apoptosis in human cancer cells [5]. The p53 phosphorylation at the residue of Ser15 in the human cell line has been linked to apoptosis which is induced by chemotherapeutic and chemopreventive agents [6]. The factor of p53 has been recognized as a transcription factor that transactivates apoptotic target genes such as Fas/CD95, death receptor 5 (DR5), BH3 interacting domain death agonist (Bid), phorbol-12-myristate-13-acetate-induced protein 1 (PMAIP 1), and Bcl-2 binding component 3 (PUMA) [6, 7]. On the other hand, upregulated p53 gene expression has been implicated in both extrinsic and intrinsic apoptotic signaling pathways [8, 9].

**Figure 1:**
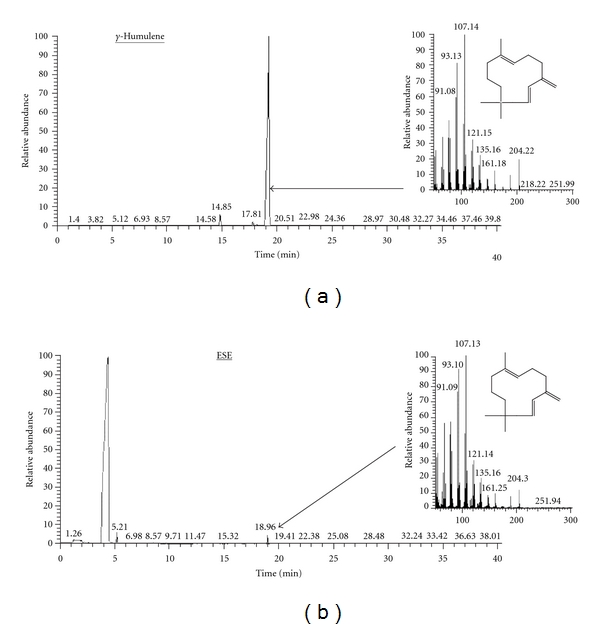
Representative GC-MS analysis of methanolic* Emilia sonchifolia* extract (ESE) and MS spectra of *γ*-humulene. The examination conditions and monitoring wavelength of GC-MS analysis were described in the profiles indicated the standard compound (*γ*-humulene) (a) and ESE (b), respectively.

*Emilia sonchifolia *(L.) DC (*Compositae*) is a folklore medicinal plant in China and Taiwan. It is used for inflammation, rheumatism, inflammation, eye sores, convulsion, cough, cuts, and wounds. In previous studies, the methanolic *Emilia sonchifolia *extract (ESE) exhibited anticancer property, anti-inflammatory, and antioxidant activity [10]. In chemical study, it is shown that the *Emilia sonchifolia* contains alkaloids, flavonoids, and terpernes, including *γ*-humulene, kaempherol-3-d-galactoside, quercitrin, quercetin, rutin, ursolic acid, senkirkine, doronine, *β*-sitosterol, and stigmasterol [11, 12]. Our earlier study has demonstrated that *γ*-humulene (Figure 1(a)) induces cell apoptosis in human colorectal cancer HT29 cells through a death receptor 5- (DR5-) mediated pathway [11]. However, there is no report addressing the possible anticolorectal cancer mechanism of methanolic ESE. The goal of this study was to investigate the molecular mechanisms of apoptosis induced by ESE in HCT 116 human colon cancer cells harboring the wild-type *p53* gene. Based on our results, we suggest that ESE induces extrinsic and intrinsic apoptotic pathways in HCT 116 cells through a p53-mediated ATM/Fas signaling.

## 2. Materials and Methods

### 2.1. Chemicals and Reagents

 Agarose, caffeine, DAPI (4,6-diamidino-2-phenylindole dihydrochloride), DMSO (dimethyl sulfoxide), *γ*-humulene, NAC (*N*-acetylcysteine), MTT (3-(4,5-dimethylthiazol-2-yl)-2,5-diphenyltetrazolium bromide), RNase A (ribonuclease-A), and Triton-X 100 were purchased from Sigma-Aldrich Corp. (St. Louis, MO, USA). RPMI-1640 medium, McCoy's 5a medium, RPMI-1640 medium, fetal bovine serum (FBS), L-glutamine, penicillin-streptomycin, and H_2_DCF-DA were obtained from Invitrogen Life Technologies (Carlsbad, CA, USA). Ccaspase-3, -8 and -9 colorimetric assay kits, Z-DEVD-FMK (a caspase-3 inhibitor), Z-IETD-FMK (a caspase-8 inhibitor), and Z-LEHD-FMK (a caspase-9 inhibitor) were purchased from R&D Systems, Inc. (Minneapolis, MN, USA).

### 2.2. Plant Material and Preparation of Methanolic *Emilia sonchifolia* Extract (ESE)

ESE was provided by Dr. Yu-Hsuan Lan (School of Pharmacy, China Medical University) and prepared as described previously [10, 13]. Briefly, this plant was dried and powdered, and then extracted with light petrol (60–80°C) and filtered with 70% methanol (Sigma-Aldrich Corp.) at room temperature. The combined methanolic extracts were filtered and evaporated under reduced pressure. The extract was resuspended in DMSO and used for *in vitro* cytotoxicity and further experiments.

### 2.3. GC-MS Analysis of Methanolic ESE

 The compositions of methanolic ESE were analyzed by GC-MS (DSQ II Single Quadrupole GC/MS, Thermofisher Scientific, USA), equipped with a 30 m × 0.25 mm × 0.25 *μ*m DB-5MS (Agilent J&W Scientific). The GC oven temperature was programmed from 60°C, held for 1 min and raised to 250°C at 4°C/min, held for 1 min, then increased by 10°C/min to 300°C, and held for 1 min to the end. The other parameters were as follows: injection temperature, 100°C; ion source temperature, 250°C; EI, 70 eV; carrier gas, He at 1.5 mL/min; injection volume, 5 *μ*L; mass range, m/z 50–1050. The identification of the major compound, *γ*-humulene, was based on a comparison of MS spectra with an authentic standard purchased from Sigma-Aldrich Corp.

### 2.4. Cell Culture

The human colorectal cancer cell line HCT 116 (wild-type p53), SW480 (p53 mutation), HT29 (p53 mutation), and human nonsmall cell lung cancer cell line A549 (wild-type p53) and H1299 (null p53) were purchased from the Food Industry Research and Development Institute (Hsinchu, Taiwan). HCT 116 cells were cultured with 90% McCoy's 5a medium. The SW480, HT29, A549, and H1299 cells were cultured with 90% RPMI-1640 medium. All media were supplemented with 10% FBS, 2 mM L-glutamine and 100 Units/mL penicillin and 100 *μ*g/mL streptomycin under a humidified 5% CO_2_, and 95% air at one atmosphere at 37°C. The medium renewal was done every 2 to 3 days [14].

### 2.5. MTT Cell Viability Assay and Morphological Observations

Cells seeded onto 96-well microplates at a density of 1 ×10^4^ cells/100 *μ*L per well were incubated with ESE at the concentrations of 0, 25, 50, 75, or 100 *μ*g/mL for a 24-hour treatment. The medium was then removed, and the cells were incubated for 3 h with 100 *μ*L of MTT solution (0.5 mg/mL MTT in PBS). The MTT-purple formazan productions were dissolved in 0.1 N isopropanol/hydrochloric acid (HCl) and optical densities of the solutions were measured by absorbance at 570 nm in an ELISA plate reader. Cell viability was expressed as the optical density ratio of the treatment to the control (% of control) as described previously [15, 16]. For determining cell morphological experiment, cells were examined and photographed using a phase-contrast microscope as described elsewhere [16, 17].

### 2.6. Assessments of Apoptosis by TUNEL Assay and DNA Gel Electrophoresis

Terminal deoxynucleotidyltransferase-mediated dUTP-biotin nick end labeling (TUNEL) assay was performed according to the manufacturer's protocols (In Situ Cell Death Detection Kit, Roche Diagnostics Corp., Indianapolis, IN, USA). Cells (1 × 10^6^/well) were plated onto six-well plates and exposed to 0, 25, 50, 75, or 100 *μ*g/mL for 24 h. After treatment, cells were collected and determined as previously described [18, 19]. TUNEL-positive cells were analyzed and quantitated using a FACSCalibur instrument (BD Biosciences, San Jose, CA, USA) equipped with BD Cell Quest Pro software. Approximately 1 × 10^6^ cells per well were incubated without (control) or with 50 *μ*g/mL of ESE for 24-hour exposure. Cells from each sample were collected and the DNA was isolated for agarose gel electrophoresis as previously described [19, 20]. After electrophoresis in a 1.5% agarose gel containing ethidium bromide (EtBr, Invitrogen) in 0.5x TBE buffer (AMRESCO Inc. Solon, OH, USA), the DNA in gel was resolved with UV light and photographed [19, 20].

### 2.7. Assays for Caspase-3, -8, and -9 Activities and Their Specific Caspase Inhibitors Pretreatment for Viability and TUNEL Assay

A density of 1 × 10^7^ cells in 75 cm^2^ flask were incubated with 50 *μ*g/mL of ESE for 0, 6, 12, and 24 h and the levels of caspase-3, -8, and -9 activities were assessed according to the manufacturer's protocol using caspase-3, -8, and -9 colorimetric assay kits (R&D Systems, Inc.). After cells from each treatment were harvested and lysed, cell lysates (50 *μ*g proteins) were incubated with caspase-3, -9, and -8 specific substrates (Ac-DEVD-pNA, Ac-LEHD-pNA, and Ac-IETD-pNA, resp.) from these kits in 96-well flat bottom microplates for 1 h at 37°C. The caspases activities were determined by measuring the release of pNA at OD_405_ using an ELISA reader (Anthos 2001, anthos Labtech Instruments, Salzburg, Austria) as previously described [16, 20]. For viability and TUNEL assay, cells were pretreated with or without the caspase-3 inhibitor (10 *μ*M; Z-DEVD-FMK), caspase-8 inhibitor (10 *μ*M; Z-IETD-FMK) and caspase-9 inhibitor (10 *μ*M; Z-LEHD-FMK) for 1 h before exposure to 50 *μ*g/mL of ESE for 24 h. Cells were harvested for determining the cell viability by MTT assay and apoptosis by TUNEL assay as described above [15, 16].

### 2.8. Real-Time PCR of Fas

Cells were cultured in 75-T flasks. ESE (50 *μ*g/mL) was added to cells for 0, 6, and 12 h. Cells were harvested and total RNA was extracted with the Qiagen RNeasy Mini Kit (Qiagen, Valencia, CA, USA). RNA samples were reverse-transcribed at 42°C with High Capacity cDNA Reverse Transcription Kit for 30 min according to the protocol of the supplier (Applied Biosystems, Foster City, CA, USA). Quantitative PCR conditions were as follows: 2 min at 50°C, 10 min at 95°C, and 40 cycles of 15 s at 95°C; 1 min at 60°C using 1 *μ*L of the cDNA reverse-transcribed as described above, 2X SYBR Green PCR Master Mix (Applied Biosystems) and 200 nM forward and reverse primers (homo Fas-F: GCAACACCAAATGCAAGAAA; homo Fas-R: GGATTCCAGATTCAGGGTCA; homo GAPDH-F ACACCCACTCCTCCACCTTT; homo GAPDH-R TAGCCAAATTCGTTGTCATACC). Applied Biosystems 7300 real-time PCR system was used for each assay in triplicate, and expression fold changes were derived using the comparative C_T_ method as previously described [17, 21].

### 2.9. Determination of Levels of Proteins Associated with Apoptotic Death and p53/Fas Signaling by Western Blotting

Cells at a density of 1 × 10^7^ cells in 75 cm^2^ flask were treated with 50 *μ*g/mL of ESE for indicated intervals of time (0, 6, 12, and 24 h or 0, 2, and 4 h, resp.). Cells at the end of each treatment period were harvested, and isolated total proteins, mitochondrial and cytosolic proteins, and protein quantification were as described previously [22, 23]. The lysates from each sample were centrifuged at 13000 ×g for 10 min and the protein concentration in the supernatant was determined with a PIERCE BCA protein assay kit (Thermo Fisher Scientific Inc. Rockford, IL, USA) as previously described [22, 24]. Equal amounts (40 *μ*g) from each sample of protein lysate were run on 10–12% sodium dodecyl sulfate-polyacrylamide gel electrophoresis (SDS-PAGE). The iBotTM Dry Blotting System (Invitrogen) was used to electrotransferred to a PVDF membrane and thereafter the blot was blocked with 5% nonfat dry milk and 0.05% Tween 20 in PBS at pH 7.4 at room temperature for 1 h. After blocking, the membranes were incubated with anti-caspase-3, anti-caspase-8, anti-caspase-9, anticytochrome *c* (Cell Signaling Technology, Danvers, MA, USA), anti-Bcl-2, anti-Bax, anti-Bid, anti-PUMA, anti-Fas, anti-FasL, anti-DR4, anti-DR5, anti-ATM, anti-p-ATM^Ser1981^ (Santa Cruz Biotechnology, Inc., Santa Cruz, CA, USA), anti-p53 and p-p53^Ser15^ (Abcam, Cambridge, U.K.) antibodies at 4°C overnight. These membranes were then incubated with horseradish peroxidase- (HRP-) conjugated goat anti-mouse or anti-rabbit IgG secondary antibodies (Millipore, Billerica, MA, USA) for 2 h at room temperature with gentle shaking. After washing, bands were visualized by Immobilon Western chemiluminescent HRP substrate (ECL) kit (Millipore) according to the manufacturer's instructions followed by development on Kodak Bio-MAX MR film (Eastman Kodak, Rochester, NY, USA). The relative abundance of each band was quantified using ImageJ software (version 1.43, NIH, USA) for Windows [17, 25]. Blots were reported with actin antibody as a loading control.

### 2.10. Immunofluorescence Staining and Confocal Laser Scanning Microscopy

Cells (5 × 10^4^ cells/well) were placed on 4-well chamber slides before being treated with 50 *μ*g/mL of ESE for 24 h. Cells were then fixed in 3% formaldehyde (Sigma-Aldrich Corp.) for 15 min, permeabilized with 0.1% Triton-X 100 in PBS for 1 h with blocking of non-specific binding sites using 2% bovine serum albumin (BSA) as described elsewhere [19, 26]. These fixed cells were stained with primary antibodies to cleaved caspase-3 (1 : 100 dilution, Cell Signaling Technology) overnight, which was detected using a FITC-conjugated goat anti-mouse IgG secondary antibody (1 : 100 dilution, green fluorescence; Millipore) followed by nuclei counterstaining using and PI (Sigma-Aldrich Corp.) (red fluorescence). Photomicrographs were obtained using a Leica TCS SP2 Confocal Spectral Microscope [26, 27].

### 2.11. Measurement of Reactive Oxygen Species (ROS) Production and *N*-Acetylcysteine, Caffeine Pretreatment for Viability

Cells at a density of 2 × 10^5^ cells/well were plated onto 12-well plates and treated with 50 *μ*g/mL of ESE for 0, 2, and 4 h followed the determinations of the changes in ROS level. Cells were harvested from each treatment, resuspended in 500 *μ*L of H_2_DCF-DA (5 *μ*M) for ROS (hydrogen peroxide; H_2_O_2_) at 37°C for 30 min. Consequently, cells were immediately analyzed by flow cytometry as described elsewhere [28, 29]. All fluorescence intensities were obtained from the mean intensity of the histogram constructed from approximately 10,000 cells using BD CellQuest Pro software. For viability assay, cells were pretreated with or without the 10 mM *N*-acetylcysteine (NAC, an antioxidant) or 1 mM caffeine (an ATM kinase inhibitor) for 1 h before exposure to 50 *μ*g/mL of ESE for 24 h. Cells were harvested for determining the cell viability by MTT assay as described above [15, 16].

### 2.12. Small Interfering RNA Transfection

Cells at a density of 2 × 10^5^ cells/well were seeded in 6-well plates and grown to 70% confluence. p53 siRNA (100 nM, Santa Cruz Biotechnology, Inc.) or control siRNA was transfected using Lipofectamine 2000 (Invitrogen) for 12 h according to the manufacturer's guideline [8]. After being transfected with p53 siRNA, cells were seeded and treated with 50 *μ*g/mL of ESE for 24-h exposure. Cells were harvested for determining the protein abundance of p53, Fas, PUMA, caspase-8, and caspase-3 by Western blotting and analysis for cell viability using MTT assay and apoptosis by TUNEL as described above [15, 16].

### 2.13. Statistical Analysis

All data were expressed as mean ± SD from at least three separate experiments. Statistical calculations of the data were obtained using Student's *t*-test with significance value of ***P < 0.001, which is considered significantly.

## 3. Results

### 3.1. Characterization of Methanolic *Emilia sonchifolia* Extract (ESE)

 Results shown in Figure 1 indicated that major composition of methanolic* Emilia sonchifolia* extract (ESE) was “*γ*-humulene” (Figure 1(a)) and the content was 63.86% (Figure 1(b)) based on peak area integrated by Thermo Xcalibur^TM^ data analysis program.

### 3.2. ESE Reduced Cell Viability and Induced Apoptotic Morphological Changes in HCT 116 Cells

**Figure 2:**
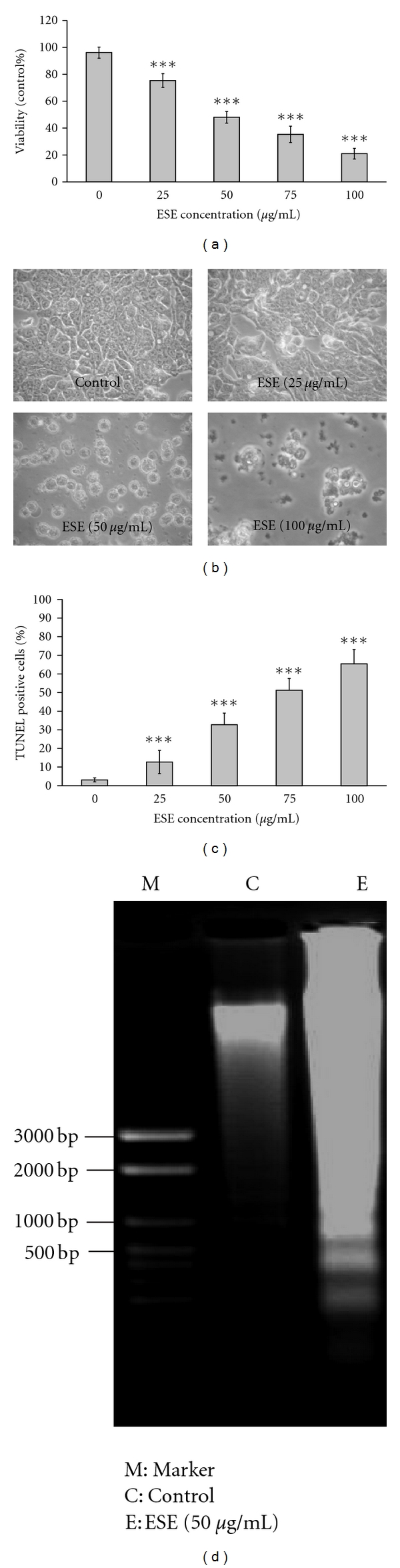
ESE reduced cell viability and induced morphological changes, apoptosis, and DNA fragmentation in HCT 116 cells. (a) Cells were exposed to various concentrations of ESE (0, 25, 50, 75, or 100 *μ*g/mL) for 24 h and determined and analyzed the viability using the MTT assay. (b) HCT 116 cells in response to 25, 50, and 100 *μ*g/mL of ESE for 24-h exposure were photographed at 200x magnification and showed apoptotic morphological changes. (c) Cells were cultured with 0, 25, 50, 75, or 100 *μ*g/mL of ESE for 24 h for determining apoptotic cells by TUNEL assay and flow cytometric analysis. (d) Treatment with ESE (50 *μ*g/mL) in HCT 116 cells shows DNA ladders by DNA gel electrophoresis. The values presented are the mean ± S.D. (*n* = 3) from three independent experiments. ***P < 0.001 shows a significant different when compared to control (0 h) sample.

 To investigate the cytotoxic responses of the ESE in HCT 116 cells, cells were exposed to various concentrations of ESE (0, 25, 50, 75 or 100 *μ*g/mL) for 24 h, and determined and analyzed the viability using the MTT assay. Results shown in Figure 2(a) indicated that ESE decreased the viable HCT 116 cells and this effect was in a concentration-dependent manner.  Figure 2(b) indicates that ESE-treated HCT 116 cells occurred rounding and shrinking, which exhibited the morphological changes when cell apoptosis. Treatment with ESE in 50 *μ*g/mL concentration, which is a close to half maximal inhibitory concentration (IC_50_) for 24 h, was used for further experiments in this study.

### 3.3. ESE Induced Apoptosis and DNA Fragmentation in HCT 116 Cells

To test whether ESE induced the decrease of cell viability and cytotoxicity contributes to apoptotic death in HCT 116 cells* in vitro*. Cells were incubated with 0, 25, 50, 75, or 100 *μ*g/mL of ESE for 24 h and then determined using TUNEL assay. Our data revealed that apoptotic evidence in ESE-treated HCT 116 cells concentration dependently increased and TUNEL-positive cells were from 14% to 63% populations after ESE treatment at 25–100 *μ*g/mL when compared to the control (0 *μ*g/mL) group (*P* < 0.05) (Figure 2(c)). Moreover, DNA gel electrophoresis confirmed that ESE induced apoptosis and DNA ladders in HCT 116 cells after 50 *μ*g/mL of ESE exposure (Figure 2(d)).

### 3.4. ESE Enhanced the Activities of Caspase-3, -8 and -9 in HCT 116 Cells

**Figure 3:**
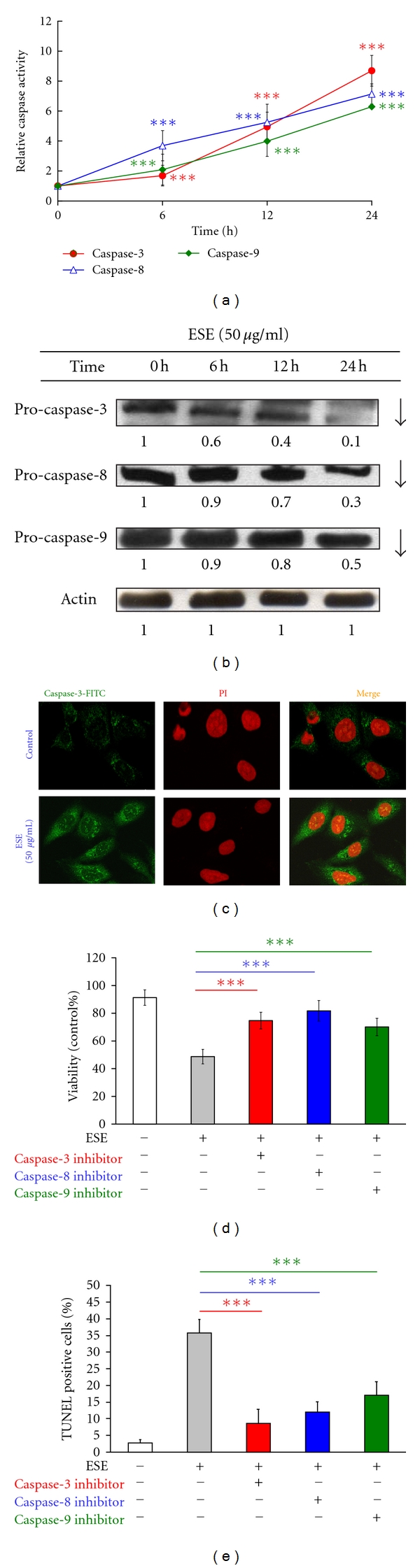
ESE affected the activities and protein levels of caspase-3, -8, and -9 in HCT 116 cells. Cells were treated with 50 *μ*g/mL of ESE for 6, 12, and 24 h. (a) ESE stimulated the activities of caspase-3, caspase-8, and caspase-9 in HCT cells as described in Section 2. The values presented are the mean ± S.D. (*n* = 3) from three independent experiments. ***P < 0.001 indicates a significant different when compared to control (0 h) sample. (b) The total proteins were harvested from ESE (50 *μ*g/mL) treated HCT 116 cells for 0, 6, 12, and 24 h and determined the protein levels of pro-caspase-3, pro-caspase-8, and pro-caspase-9 by Western blotting. Actin was used as a loading control. (c) ESE stimulated the translocation of caspase-3 trafficking to nuclei in HCT 116 cells by confocal laser scanning microscope as described in Section 2. Cells were pre-incubated with or without specific inhibitors of caspase-3 (Z-DEVD-FMK), caspase-8 (Z-IETD-FMK), and caspase-9 (Z-LEHD-FMK), respectively, and then treated with ESE-(50 *μ*g/mL) for 24 h. The cellular viability was assessed by MTT assay (d) and apoptotic cells were assessed by TUNEL assay and flow cytometric analysis (e). The values presented are the mean ± S.D. (*n* = 3) from three independent experiments. ***P < 0.001 shows a significant different when compared to ESE treatment.

 The observation of apoptotic death induced by ESE raised the possibility that activations of caspase cascades were required for examining the treated HCT 116 cells* in vitro*. Thus, we next examined the proteolytic activation of caspase cascades in ESE-treated HCT 116 cells and our results indicated that ESE stimulated caspase-3, caspase-8, and caspase-9 activities in a time-dependent effect (Figure 3(a)). The protein levels of pro-caspase-3, pro-caspase-8, and pro-caspase-9 were downregulated in HCT 116 cells after ESE exposure (Figure 3(b)). In addition, the result in Figure 3(c) from confocal microscopy indicated ESE stimulated the translocation of caspase-3 trafficking to the nuclei when compared to the control sample. Importantly, pretreatment with specific inhibitors of caspase-3 inhibitor (Z-DEVD-FMK), caspase-8 inhibitor (Z-IETD-FMK), and caspase-9 inhibitor (Z-LEHD-FMK), respectively, significantly prevented against the ESE-induced cell growth inhibition (Figure 3(d)) and apoptosis in HCT 116 cells (Figure 3(e)).

### 3.5. ESE-Triggered Apoptosis in Mediated Mitochondria- and Death-Receptor-Dependent Signaling in HCT 116 Cells

**Figure 4:**
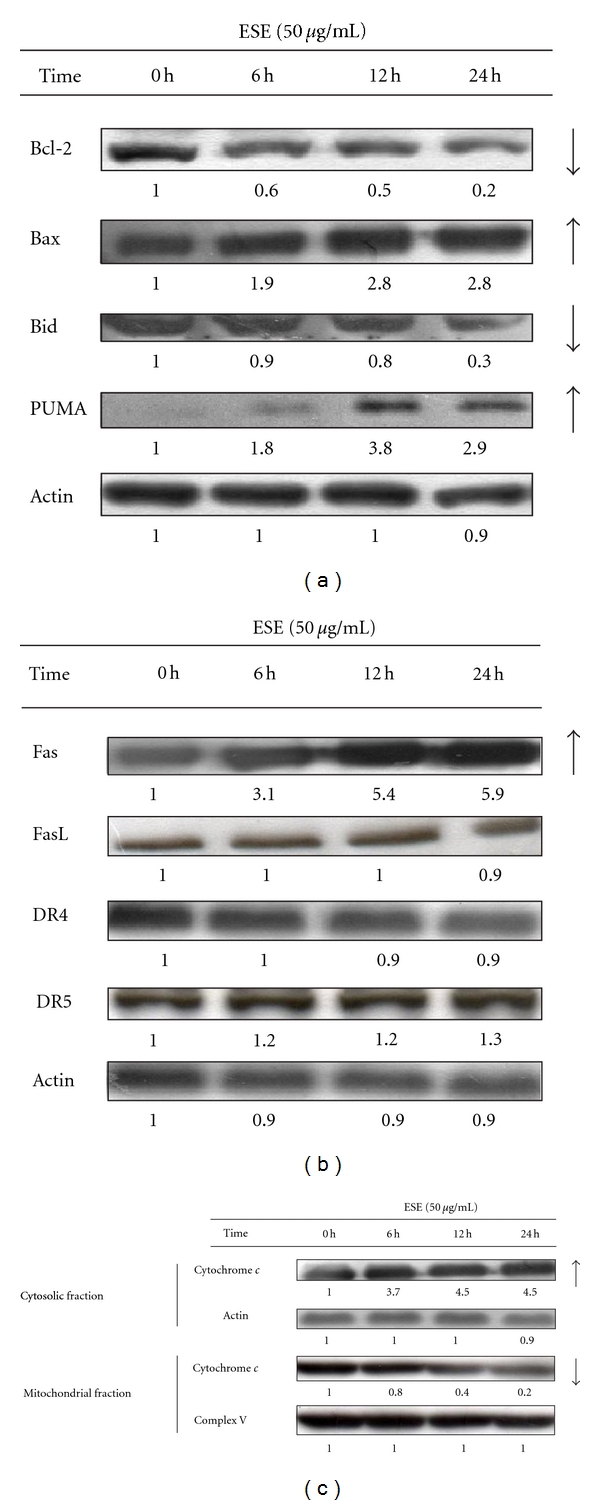
ESE altered the protein abundance-associated with mitochondria- and death-receptor-dependent apoptotic signaling in HCT 116 cells. Cells were treated with ESE (50 *μ*g/mL) for 0, 6, 12, 24 h, and total protein, cytosolic, and mitochondrial lysates were prepared and subjected to Western blotting analysis. The membranes were incubated with (a) anti-Bcl-2, anti-Bax, anti-Bid and anti-PUMA antibodies; (b) anti-Fas, anti-FasL, anti-DR4 and anti-DR5 antibodies; (c) anticytochrome* c* antibody. The blot was also probed with anti-Actin and anti-Complex V antibodies to confirm equal loading of samples. Each band was quantified using ImageJ software.

To assess the alterations in apoptosis-related protein levels in ESE-treated HCT 116 cells, we administered ESE at the concentration of 50 *μ*g/mL for 0, 6, 12, and 24 h in HCT 116 cells and then evaluated the protein levels by Western blot analysis.  Figure 4(a) shows that ESE promoted a decrease of Bcl-2 level (an antiapoptotic protein) and the increases of proapoptotic protein levels of Bax and PUMA in HCT 116 cells. Also, treatment of ESE showed that the level of Bid was downregulated in HCT 116 cells (Figure 4(a)). As shown in Figure 4(b), ESE enhanced the death receptor pathway-associated protein levels (Fas, DR4 and DR5) in HCT 116 cells. Alternatively, the level of cytochrome *c *from cytosolic fraction is upregulated and from mitochondrial fraction is downregulated in ESE-treated HCT 116 cells (Figure 4(c)). Collectively, these results suggest that both intrinsic (mitochondria) and extrinsic (death-receptor-) dependent pathways contributed to ESE-provoked apoptotic death in HCT 116 cells.

### 3.6. ESE Increased ROS Production and Stimulated ATM/p53/Fas Signaling in HCT 116 Cells

**Figure 5:**
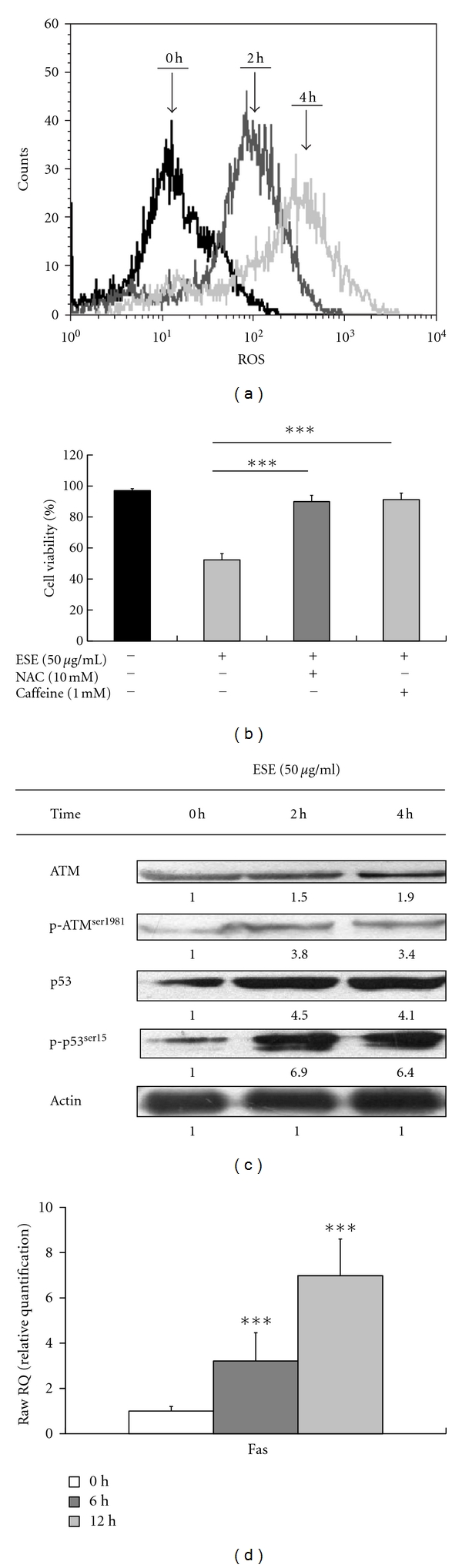
ESE increased ROS production and contributed to p53-correlated ATM/Fas apoptotic signaling in HCT 116 cells. (a) Treatment with ESE (50 *μ*g/mL) for the indicated times (0, 2, and 4 h) was subjected to ROS productions by flow cytometry as described in Section 2. (b) Pretreatment with NAC (10 mM, a scavenger of ROS), or caffeine (1 mM, an inhibitor of ATM) in ESE-treated HCT 116 cells restored the cell viability by MTT assay. The values presented are the mean ± S.D. (*n* = 3) from three independent experiments. ***P < 0.001 shows a significant different when compared to ESE treatment. (c) ESE elevated the protein levels of ATM, phosphorylated ATM (Ser1981), p53, phosphorylation, and p53 (Ser15) by Western blotting. (d) Effects of ESE on Fas mRNA level in HCT 116 cells, and the total RNA was extracted from each treatment of ESE (50 *μ*g/mL) on HCT 116 cells for 0, 6, and 12 h. RNA samples were reverse transcribed into cDNA and quantified with real-time PCR as described in Section 2. The ratios of Fas mRNA/GAPDH are presented. The values presented are the mean ± S.D. (*n* = 3) from three independent experiments. ***P < 0.001 shows a significant different when compared to control (0 h) sample.

Results shown in Figure 5(a) revealed that ESE promoted the level of intracellular ROS production (2 h treatment: 81.26 ± 2.39%; 4 h treatment: 90.34 ± 4.18%) in HCT 116 cells by flow cytometry and a specific fluorescent probe, H_2_DCFDA for determining ROS level. Cells pretreated with *N*-acetylcysteine (NAC, an antioxidant) and caffeine (an ATM kinase inhibitor) significantly reduced ESE-induced growth inhibition effect (Figure 5(b)). Previous studies have stated that p53 gene and its phosphorylation at the Ser15 interacted Fas/CD95 activation when cell apoptosis occur [7, 8, 30]. To elucidate the crucial roles of ATM, p53 and Fas in HCT 116 cells after treatment with ESE, the protein levels of ATM, p-ATM^Ser1981^, p53, and p-p53^Ser15^ expression were investigated by Western blot analysis. Our results showed that ESE increased the protein levels of ATM, p-ATM^Ser1981^, p53 and p-p53^Ser15^ in HCT 116 cells as can be seen in Figure 5(b). Our further study investigated if p53 affects the Fas expression in ESE-treated HCT 116 cells. We hypothesized that ESE induces apoptosis through the increase of Fas/CD95 by p53-dependent transcriptional activation. real-time PCR analysis was performed to determine whether the induction of Fas/CD95 protein level by ESE was due to increased the level of mRNA. As shown in Figure 5(d), the 6 and 12 h treatment of HCT 116 cells with ESE (50 *μ*g/mL) led to an increase in mRNA levels of Fas/CD95. Our results indicate that ESE increased the protein level of Fas/CD95 through the p53-dependent regulation of transcription levels.

**Figure 6:**
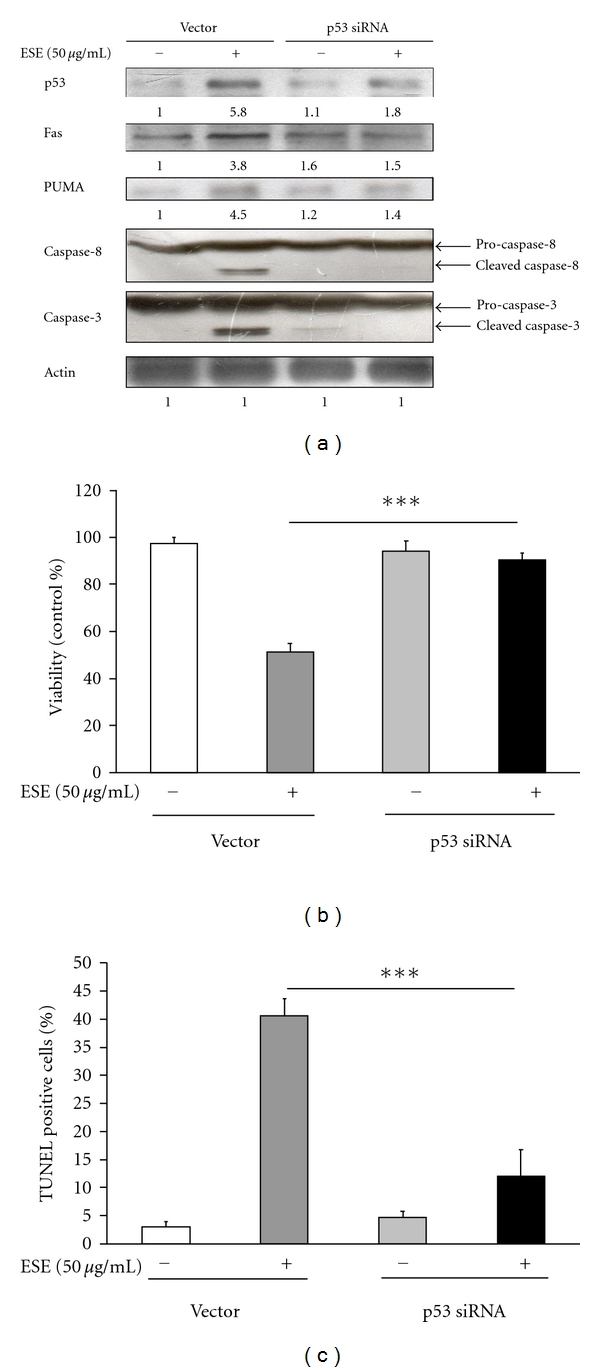
ESE-affected cytotoxicity and apoptosis is mediated through alterations of p53 downstream signals in p53 siRNA-transfected HCT 116 cells. (a) The p53 siRNA or control siRNA-transfected HCT 116 cells were treated with ESE (50 *μ*g/mL) for 12 h, and total protein was prepared and subjected to Western blotting analysis. The membranes were incubated with anti-p53, anti-Fas, anti-PUMA, anticaspase-8 and, anti-caspase-3 antibodies. The blot was also probed with anti-Actin antibody to confirm equal loading of samples. Each band was quantified using ImageJ software. The p53 siRNA or control siRNA-transfected HCT 116 cells were treated with ESE (50 *μ*g/mL) for 24 h, cell viability was determined by MTT assay (b) and apoptotic cells were assessed by TUNEL assayand flow cytometric analysis (c). The values presented are the mean ± S.D. (*n* = 3) from three independent experiments. ***P < 0.001 shows a significant different when compared to control sample.

Therefore, our results showed that the induction of p53, Fas, PUMA, cleaved caspase-8, and cleaved caspase-3 due to ESE treatment was correlated with the decrease in p53, Fas, PUMA, cleaved caspase-8, and cleaved caspase-3 protein levels by the transfection with p53 siRNA in HCT 116 cells (Figure 6(a)). We found that ESE-reduced viability in HCT 116 cells was nearly enhanced after using p53 siRNA compared to the ESE alone sample as shown in Figure 6(b). We also found that ESE induced apoptosis in HCT 116 cells was nearly prevented after using p53 siRNA compared to the ESE alone sample as shown in Figure 6(c). Taken together, these results suggest that p53 activation is an important factor in ESE-induced apoptosis of HCT 116 cells, which is mediated through ROS productions (oxidative stress) and ATM/p53/Fas-dependent signaling pathways.

## 4. Discussion

Phytochemicals affect intracellular targets, and this characteristics often makes desirable on tumor cells as chemopreventive or chemotherapeutic agents against cancer [31]. The previous study showed that ESE inhibited lymphoma, Ehrlich ascites carcinoma, and mouse lung L-929 fibroblast cells, and it is important that ESE is not toxic to normal cells *in vitro *[10]. *In vivo* study also indicated that oral administration of the ESE (100 mg/kg body weight) to mice increased the life span and reduced the solid tumor volume of tumor-bearing mice [10–12]. In the present study, we firstly demonstrated that ESE reduced cell proliferation in HCT 116 human colorectal cancer cells through induction of cell apoptosis. Additionally, it had low toxicity to human normal skin fibroblast Detroid 551 cells (IC_50_ > 200 *μ*g/mL) *in vitro* (data not shown). The IC_50_ for 24 h treatment of ESE in HCT 116 and HT29 cells were 50.54 ± 2.28 and 88.54 ± 4.01 *μ*g/mL, respectively (Table 1). Because p53 is frequently expressed in colorectal cancer, new agents that preferentially kill p53-expressing cells are highly desirable chemotherapeutic agents. To investigate the selective toxicity of ESE in different p53 gene expression cell lines, we demonstrated three human colorectal cancer cell lines (HCT 116, SW480, and HT29) and two lung cancer cell lines (A549 and H1299) [32–35].

The IC_50_ values calculated from these results are reported in Table 1; SW480, HT29, and A549 cell lines, which carries a mutant form of the *p53 *gene, are significantly low cytotoxic action of ESE than the HCT116 cell line, carrying a wild-type *p53 *gene. However, H1299 cells are 2-fold IC_50_ values to ESE than HCT116 cells (Table 1). In comparison to the mutant p53 cell lines or null p53 cell line, a greater cytotoxic effect was found in the wild-type p53 cell lines. The results suggested that ESE preferentially induced more cytotoxic effect in the wild-type p53 lines than in the mutant or null p53 colorectal cancer cells. The reasons for the differences in sensitivities in IC_50_ of those cell lines may be due to the intrinsic different* p53* gene in different types of cell lines. The p53 in SW480 and HT29 cells has been shown to be a mutated gene with a mutation at codon 273 and that in HCT 116 cells is future to be functional without mutation [36]. It is reported that p53 is a mediator of chemotherapy-induced cell death, resulting from ROS productions which is activated by chemotherapeutic agents [37]. Many studies reported that cisplatin did not significantly increase apoptosis in p53-mutant cells, but a significant increase in the apoptotic index was observed in wild type p53 cells which correlates with increased p53 protein level [38–40]. Our results suggest that ESE induced apoptosis in HCT 116 cells through p53-mediated signaling. In this study, our results demonstrated that *γ*-humulene is the major constituent in *Emilia sonchifolia* by GC/MS analysis (Figure 1). Recently, we first demonstrated that *γ*-humulene has anticancer activity by stimulating the clustering of DR4/DR5 and associated FADD protein levels, leading to caspase-8 and caspase-3 activation, and then induction of apoptosis in HT29 cells [11]. Correctively, our results suggest that *γ*-humulene is the major bioactive compound in ESE.

Apoptosis is an intracellular suicide program possessing morphologic change and biochemical response. In the present study, we showed that ESE reduced the cell viability in HCT 116 cells in a concentration-dependent manner (Figure 2(a)) and triggered apoptotic morphological changes (Figure 2(b)). ESE induced DNA condensation and fragmentation by DNA gel electrophoresis and TUNEL staining (Figures 2(c) and 2(d)). Two major apoptotic pathways have been described the extrinsic (death receptor mediated) and the intrinsic pathway (mitochondria mediated) [41]. The intrinsic apoptotic pathway affected mitochondrial permeability, releases cytochrome *c*, Apaf-1, Endo G, and pro-caspase-9 proteins from mitochondria to cytosol, leading to activation of caspase-9. The extrinsic apoptotic pathways originateing at membrane death receptors include Fas/CD95, DR4 and DR5 and then influence the intracellular apoptotic adaptor FADD protein and proximal caspase-8 as well as distal executioner caspases [41–43]. Our results demonstrated that ESE significantly increased activities of caspase-3, caspase-8, and caspase-9 (Figure 3(a)) after 6 to 24 h treatment, and pretreatment with specific inhibitors of caspase-3, -8, and -9, respectively, led to increased viable cells in ESE treatment (Figure 3(c)). On the other hand, pretreatment of cells with specific inhibitors to caspase-3, -8, and -9 significantly prevented the ESE-induced cell apoptosis (Figure 3(e)). Our results suggest that both of extrinsic and intrinsic apoptotic pathways may be involved in the ESE-provoked apoptotic death in HCT 116 cells.

Recently, many studies have shown that p53-inducible proapoptotic genes trigger apoptosis through both extrinsic and intrinsic apoptotic pathways [44–46]. In this study, we demonstrated that the ESE significantly increased ROS production (Figure 5(a)), the protein levels of PUMA (Figure 4(a)), Fas (Figure 4(b)), ATM, p-ATM^Ser1981^, p53, p-p53^Ser15^,and in HCT 116 cells (Figure 5(b)). In addition, knockdown of p53 expression by p53 siRNA significantly inhibited the protein level of Fas/CD95, PUMA, cleaved caspase-8 and cleaved caspase-3 (Figure 6(a)), cell growth-inhibitory effects (Figure 6(b)), and apoptosis (Figure 6(c)) after treatment with ESE in HCT 116 cells. According to our results, we suggest that p53 might be involved in ESE upregulated Fas-mediated extrinsic apoptotic signaling pathways.

**Figure 7:**
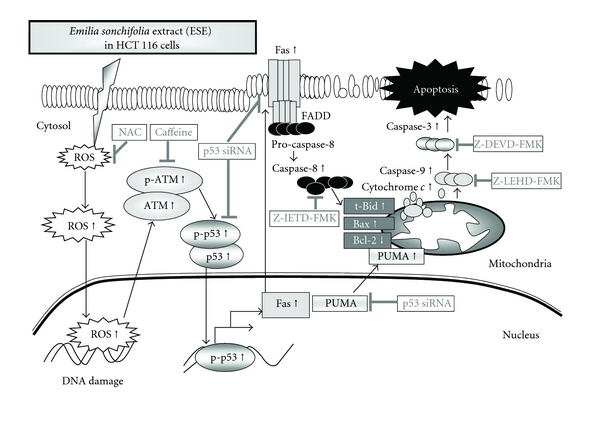
A proposed working model for the action and possible signaling pathways of ESE on HCT 116 human colorectal cancer cells. ESE induces apoptosis through both extrinsic and intrinsic apoptotic pathways, resulting from p53-mediated ATM/Fas signaling, which counteracts the induction of apoptotic death in HCT 116 cells (see text for details).

In conclusion, the molecular signaling pathways are summarized in Figure 7. The present study revealed that ESE (i) decreased the percentage of viable cells; (ii) induced apoptotic morphological changes and DNA fragmentation; (iii) upregulated the protein levels and activated the levels of caspase-3, caspase-8, and caspase-9; (iv) increased the ROS production and upregulated the protein levels of ATM, p-ATM^Ser1981^, p53 and p-p53^Ser15^; (V) stimulated the p53 downstream protein levels of Fas/CD95 and PUMA. Our results suggest that ESE warrants further development as a colorectal cancer prevention or therapeutic agents in the future.

## Figures and Tables

**Table 1:** The IC_50_ values for 24 hour-treatment of ESE in different *p53* gene expression cell lines (mean ± S.D. of three independent experiments).

Cell line		*p*53 gene expression	IC_50_ (*μ*g/mL)
HCT 116	Human colorectal cancer cell lines	Wild-type	50.54 ± 2.28
SW480		Mutation (Arg^273^→His)	79.62 ± 6.05
HT29		Mutation (Arg^273^→His)	88.54 ± 4.01
A549	Human nonsmall cell lung cancer cell lines	Wild-type	53.69 ± 3.17
H1299		Null	121.36 ± 5.33
